# Exploring mechanisms of behavior change for healthcare professionals in cough and secretion management in ALS

**DOI:** 10.1080/17582024.2025.2506954

**Published:** 2025-05-20

**Authors:** Charlotte Massey, Esther Hobson, Alys Wyn Griffiths, Lucy Musson, Christopher McDermott

**Affiliations:** a Sheffield Institute for Translational Neuroscience, University of Sheffield, Sheffield, UK; b Department of Neurology, Sheffield Teaching Hospitals NHS Foundation Trust, Sheffield, UK

**Keywords:** Motor neuron disease (MND), amyotrophic lateral sclerosis (ALS), cough, sialorrhoea, secretion management, multidisciplinary care

## Abstract

**Objectives:**

To explore healthcare professionals’ experiences managing cough and secretion problems in Amyotrophic Lateral Sclerosis (ALS).

**Methods:**

A qualitative study was completed with 23 individuals participating in four focus groups. Data was analyzed using reflexive thematic analysis and COM-B and theoretical domains framework (TDF) behavior change frameworks.

**Results:**

This study found that roles, responsibilities, and expectations needed to be clearly defined and that building relationships was important to support care delivery. Barriers identified included limited access to specialist care, equipment, and opportunities to gain knowledge and skills. A structured clinical assessment was highlighted to enable good-quality care. Data mapped most commonly to the environmental context/resources, knowledge, skills (TDF), and physical capability (COM-B) behavior change domains.

**Conclusion:**

Cough and secretion management in ALS is complex due to the multifaceted nature of the disease. This study emphasizes the need for future development of clinical interventions to support care.

## Plain Language Summary

People living with Amyotrophic Lateral Sclerosis (ALS) frequently have issues with breathing, swallowing, drooling, and cough weakness. There is limited existing knowledge to tell healthcare professionals how to manage these problems and to date there is no research looking at healthcare professionals’ experiences managing these. This study aimed to understand how healthcare professionals in the UK manage cough and secretion problems in people with ALS, what challenges they face and how care can be improved.

Four focus groups were completed with 23 healthcare staff. The study found that the roles and responsibilities of healthcare staff needed to be clearly defined and that building relationships was important to support care. Several barriers were identified such as limited access to specialist care, equipment, and opportunities to gain knowledge and skills. Understanding the key problems was noted to be key to providing the right cough and secretion management for people with ALS. We concluded that managing these issues in ALS is complicated as the disease is variable. To provide the best care to people living with ALS we need to develop new tools to support healthcare staff to make decisions around treatment.

## 1. Introduction

Amyotrophic Lateral Sclerosis (ALS) is a rapidly progressive neurological condition caused by upper and lower motor neuron degeneration. There is currently no cure. Interventions are used to compensate for the progressive loss of motor, respiratory, and swallow function and support quality of life, with only a limited impact on increased survival [1,2]. Respiratory impairment is the leading cause of mortality and morbidity in ALS [2]. Respiratory dysfunction in ALS can be characterized by a progressive decline in ventilatory function [2], a reduced ability to cough and therefore expectorate respiratory and oropharyngeal secretions [3] and aspiration of secretions and/or food and drink due to dysphagia [4]. ALS can be categorized into different types depending on the site of onset [5]: Bulbar onset, limb (classic) onset, predominantly upper motor neuron, flail limb onset, or respiratory onset.

Cough and secretion issues in ALS are multifactorial and impact negatively on quality of life including sleep and social participation [6]. Up to 70% of people with ALS will have issues with managing saliva and secretions [7], and this is more prevalent in those with bulbar onset ALS. Additionally, the presence of upper airway secretions is the biggest predictor of poor tolerance of noninvasive ventilation (NIV), one of the few available treatments which improves survival and quality of life [2,8]. There is a known interdependent relationship between the upper airway and respiration, and recent research highlights the key relationship between the upper airway and the lower airways in ALS [9–17]. Cough and secretion management in ALS usually involves an airway clearance technique to augment cough strength due to respiratory muscle weakness [18,19], pharmacological management of oropharyngeal and respiratory secretions and/or other treatments such as optimizing posture and positioning or orthotics [20]. Airway clearance techniques can be classed as proximal techniques (cough augmentation), such as Mechanical Insufflation-Exsufflation (MI-E) or Lung Volume Recruitment (LVR) [21], or peripheral techniques, such as manual techniques or High Frequency Chest Wall Oscillation (HFCWO). These can be used in isolation or combined [22]. Individualized cough augmentation programs and MI-E settings are advised in ALS due to the known bulbar impairments [10,23] and protocols for the initiation of individualized MI-E have been shared in the literature [10,24,25], however there is no standardized protocol for LVR [26]. New ways to evaluate the efficacy of MI-E are emerging, including the use of MI-E waveforms [27,28] and direct visualization of the upper airway [10,11], and translaryngeal ultrasound [28]; however, these are still rarely seen within clinical practice. Symptomatic therapy requires a fine balance of treatments: for example, treating sialorrhea and the accompanying side effects such as thick secretions and dry mouth.

The existing guidelines in cough and secretion management [20,29,30] rely on low-quality evidence and expert consensus and treat cough, secretion management, and ventilatory failure as separate entities. These guidelines do not account for the ways that these impact on each other, and how this subsequently affects treatment. The broad statements in these guidelines do not guide clinicians to make decisions around assessment techniques and analysis of these to support treatment, meaning clinicians are often unsure on the best course of treatment.

ALS care in the UK is provided by the National Health Service (NHS) via a network of specialist care centers or networks. These specialist centers offer diagnosis services and provision of specialist interventions such as NIV and MI-E, but are often situated in large cities. They are frequently many miles away from patients’ homes, meaning they are difficult to access. Subsequently, day to day care is mostly provided by community services, who may not have specialist ALS knowledge or skills. It is well documented that multidisciplinary care improves survival and quality of life in ALS [6,31,32]; however, this successful model is often not applied when managing people with cough and secretion symptoms [33,34]. Within the UK healthcare system, these issues are often assessed and managed in silos by different professionals and different healthcare teams. Airway clearance techniques are primarily issued by specialist respiratory physiotherapists, training people with ALS and their caregivers how to use these on a day-to-day basis. Cough augmentation devices (such as MI-E and LVR devices) are widely available on the NHS, however the criteria for accessing and the wait to access these may differ by area. Secretion management strategies, however, involve numerous healthcare professionals [35]. These large extended multidisciplinary care teams, made up of community and ALS center staff, may find it difficult to provide consistent care and support with complex decision-making [36].

The complex and multifaceted nature of cough secretion management is often highlighted [37]; however, there were no studies looking at the experiences of healthcare professionals managing these within ALS care. A survey of UK healthcare professionals managing cough and secretion problems in ALS [35] highlighted that they felt under confident in assessing cough and secretion problems and recommending and implementing treatment plans. The reasons for this remain unknown, so this study aimed to contextualize the survey findings [35] using focus group discussions with UK healthcare professionals to identify the barriers and enablers of care and consider how care could be improved.

The COM-B (Capability, Opportunity, Motivation, Behavior) model of behavior change was used as an overarching model to interpret the key barriers and enablers to care. This model aims to understand the determinants of a specific behavior, using six components which all interact and influence each other [38]. The Theoretical Domains Framework (V2) (TDF) elaborates on the COM-B components and has 14 domains. It gives a detailed analysis of a multitude of factors; environmental (e.g., resources available), social (interpersonal influences), cognitive (decision-making), and affective (optimism and intentions) [39]. Table 1 summarizes the components/domains of COM-B and TDF.

**Table 1.** Components/Domains of COM-B and TDF.

COM-B component	Theoretical domains framework
Psychological capability	Knowledge
	Memory, attention and decision processes
	Behavioural recognition
Physical capability	Skills
Social opportunity	Social influences
	Reinforcement
Physical opportunity	Environmental context/resources
Reflective motivation	Social professional role and identity
	Beliefs about capability
	Optimism
	Beliefs about consequences
	Intentions
	Goals
Automatic motivation	Emotion

COM-B = Capability, opportunity, and motivation behavior model, TDF = Theoretical Domains Framework.

These frameworks have been used to support behavior change analysis implementations within healthcare settings [39–42] and within the context of ALS [43]. COM-B and TDF can be used alongside the behavior change wheel to identify ‘the means by which an intervention can change behaviour’ [44]. Within this study, these theoretical frameworks were used together to support identification of behaviors and factors that could be targeted in the development of future cough and secretion interventions.

### 1.1. Aims

The present study aimed to explore:

1. Experiences of healthcare professionals providing cough and secretion management to people with ALS in the UK.
2. Barriers and enablers of good-quality cough and secretion management from healthcare professionals’ perspectives.
3. Ways to optimize care for people with ALS who have cough and secretion issues.

## 2. Materials and methods

### 2.1. Study design

A qualitative approach consisting of a series of focus groups was used to explore the perceptions of UK healthcare professionals. The focus group topic guide was informed by the preliminary findings from our previously published survey [35]. Borton’s ‘What?,’ ‘So what?’ and ‘Now what?’ framework [45] was used to structure the focus groups. This encouraged participants to reflect on existing findings from the survey in relation to their own experiences and clinical practice, the implications of these findings, and what should be done or changed going forward in practice. The structure of the focus groups can be seen in Table 2.

**Table 2.** Structure of the focus groups using Borton’s framework.

Borton’s framework	Focus group plan
What?	Participants were presented initial findings from the survey [35] at the beginning of the group and asked to look through these. They were asked to consider these in relation to their own clinical practice and experience to facilitate discussion (supplementary material 1).
So what?	Considering the survey findings at the level of the patient, the service and the professional to discuss barriers and enablers of care.
Now what?	A discussion about how the identified enablers and barriers and ways in which changes in clinical practice could improve care.

Topic guides were developed without alignment to TDF domains so that participants’ responses were not constrained to specific domains [46]. They were piloted with a group of ALS specialists and refined. The COREQ checklist was used to ensure quality of reporting (supplementary material 2).

### 2.2. Sample and sampling

All UK healthcare professionals involved in cough and secretion management for people with ALS were eligible to participate. Participants were recruited using social media, charity partners such as the UK MNDA and special interest groups such as the Association of Physiotherapists in Respiratory Care (ACPRC). The MNDA ran two workshops on cough and secretion management in autumn 2023 and all attendees were offered an opportunity to join a focus group before the workshop. Two focus groups were run face-to-face in London and Newcastle and two were completed virtually to allow a geographical spread of participants.

### 2.3. Data collection and analysis

Data was collected between November 2023 and January 2024. Discussion was co-facilitated by CM (an experienced physiotherapist) and LM (an experienced qualitative researcher), audio/video-recorded and transcribed verbatim, and checked for accuracy. Field notes were taken by facilitators during the groups. Pseudonyms were used to maintain participants’ data confidentiality. Participants were asked to share any resources discussed in the groups via e-mail, and any extra information received via e-mail after the group was added to the bottom of the transcript for analysis. Data was initially analyzed inductively using the six phases of reflexive thematic analysis [47,48]. Firstly, researchers familiarized themselves with the dataset. Initial coding was then completed for each transcript by CM, AG, and EH. These codes were then analyzed to look for shared meanings and initial themes were generated, which were then reviewed and defined. Development and refinement of the themes continued throughout the data analysis, and these were reported illustratively and then analytically until the final names of the themes were agreed by all three researchers (Figure 2). The data was then deductively coded by CM and LM using the six COM-B components and 14 TDF domains using manual analysis in the following way: initial coding, mapped to COM-B components, mapped to TDF domains, identification of intervention functions from the behavior change wheel, and possible behavior change techniques (Table 3 and supplementary material 3).

**Table 3.** How themes mapped to COM-B and TDF behavior change frameworks.

Theme	COM-B constructs	TDF domains
Responsibility as an enabler	Automatic motivation, reflective motivation, social opportunity, psychological capability	Knowledge, behavioral recognition, social influences, social professional role and identity, intentions, emotion and memory, attention and decision processes
Access as a barrier	Reflective motivation, physical opportunity and physical capability	Skills, environmental context/resources, intentions
Relationships as an enabler	Physical opportunity, reflective motivation, social opportunity and psychological capability	Behavioral recognition, social influences, social professional role and identity, environmental context/resources, beliefs about capacity and memory, attention and decision processes
Expectations as a barrier	Automatic motivation, physical opportunity, reflective motivation, social opportunity and psychological capability	Knowledge, social influences, social professional role and identity, environmental context/resources, beliefs about capacity, optimist, goals and emotion
Comprehensive clinical assessment as an enabler	Automatic motivation, physical opportunity, reflective motivation, social opportunity, physical capability and psychological capability	Knowledge, skills, social influences, social professional role and identity, environmental context/resources, beliefs about capacity and emotion

COM-B = Capability, opportunity, and motivation behavior model, TDF = Theoretical Domains Framework.

### 2.4. Ethics

The study received ethical approval from the University of Sheffield ethics panel (reference: 053871).

## 3. Results

**Figure 1.**
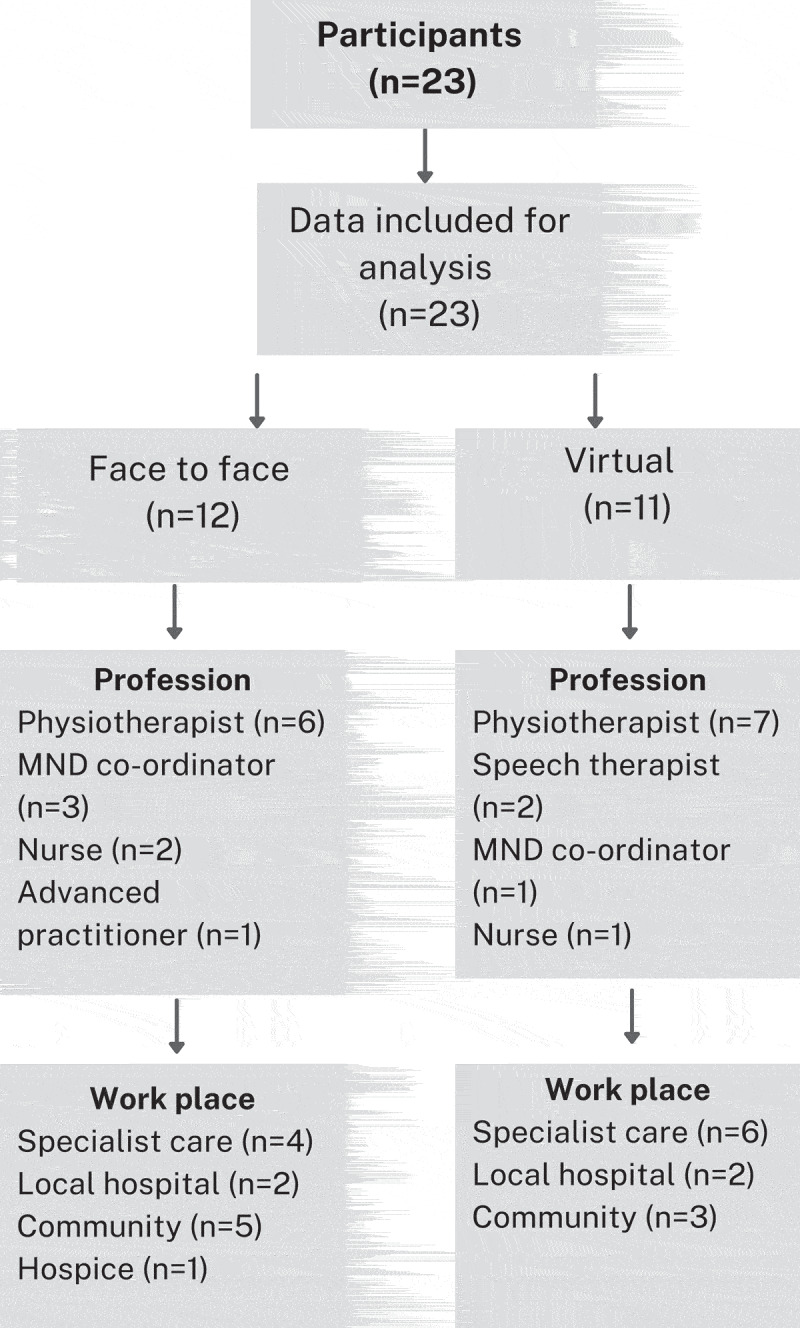
n = number of participants.

Four focus groups were conducted with 23 participants, 5–7 participants per group (see Figure 1 for demographics) and ranged from 42 to 60 min. Participants were from a range of professionals with physiotherapists making up over half of the participants (56%). Most participants worked in specialist centers (43%) or in the community (34%). All but one participant (95%) was employed by the NHS, with the remaining participant employed by a hospice, 91% (*n* = 21) were female and 91% (*n* = 21) defined their ethnicity as white British.

**Figure 2.**
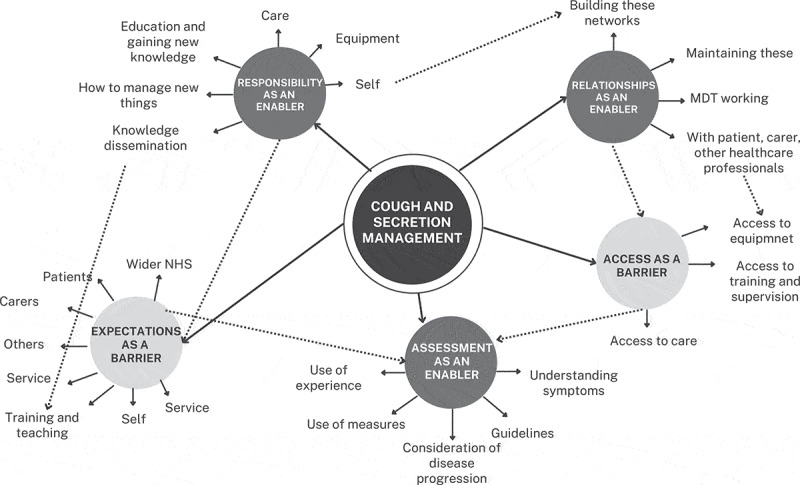
MDT = multidisciplinary team, NHS = national health service.

Five overarching themes were identified, which were then classed as barriers or enablers to care: Responsibility, access, relationships, expectations, and clinical assessment (Figure 2). Many of these could be defined as both a barrier and an enabler, but they were classed according to how they were described by participants in the focus groups. Themes were defined and are described below, linked to COM-B components and TDF domains. Table 3 and supplementary material 3 show how themes were mapped to these behavior change frameworks.

### 3.1. Responsibility as an enabler

Participants held different responsibilities, and levels of responsibility, in delivering care. Responsibilities could be both an enabler and a barrier to care, however where these were appropriate and clearly defined, they acted as an enabler of care. This included responsibility for aspects of clinical care, supervision and training of others, personal upskilling, and training and education of people with ALS and their caregivers in cough and secretion management techniques.

It almost sounds like for really effective management, we all as professionals need to know what our roles and responsibilities are. {Speech therapist 1}

Defining responsibilities when implementing service change or new technologies was highlighted as important and participants noted that responsibilities need to be clearly defined with standard operating procedures in place to ensure staff are supported to ensure the implementation is successful.

Healthcare professionals with specialist skills in cough and secretion management reported feeling that in order to have good consistent, clinical care, they felt responsible for providing constant indirect clinical supervision for less experienced staff members, which was deemed to be impractical. There was a feeling that responsibility has changed recently with healthcare professionals being more aware of governance and managing internal risk which all impact service responsibilities including direct and indirect supervision of staff.

If one of us wasn’t in that’s when someone would really struggle in the management of these patients … they could provide safe care but it certainly wouldn’t be specialist care. {Respiratory physiotherapist 1}

This was especially highlighted during an acute admission to a local hospital with a respiratory infection where responsibility for respiratory care can be unclear. Roles and responsibilities can be well defined within a single hospital structure, however these become blurred when treatment is being delivered by staff across many organizations who may be unfamiliar with each other. This is confounded by the fact that many people with ALS will be unable to share this with different healthcare providers due to dysarthria and/or cognitive impairment. The complexity of ALS care was highlighted and that it does not fit neatly within one specialty. Piecing together all of the different aspects of care can often be challenging when there is a lack of understanding about the underlying condition.

The big lack of understanding around the actual ALS in itself. The respiratory team are very good at treating the acute chest infection that’s there. But maybe not looking at the, the wider picture of those patients. {Respiratory physiotherapist 2}

Community therapists also reported feeling isolated as they were expected to take the responsibility of managing complex cough and secretion issues at home in between specialist center visits.

I feel quite uncomfortable being the sole practitioner holding that risk. I guess it is the GP [who ultimately hold the risk] but they often don’t know the person clinically. {ALS coordinator 1}

Participants felt that the service organization impacted responsibilities. It was highlighted that services assume different levels of responsibility for clinical care provision depending on where they are and the staffing, equipment, and skills they have access to. There were divided opinions about where the responsibility for education and training should lie; however, it was agreed that healthcare professionals assuming responsibility for this can lead to improved care. Many felt that it was the responsibility of specialist services to support the upskilling of nonspecialist services but the need to upskill is often identified and led by individuals rather than the organization.

There were numerous different methods for training and assessing individuals’ competence on devices such as cough assists. All mentioned the importance of considering informal carer support and patient physical abilities when providing training. It was highlighted that some NHS trusts do not allow for staff to train external formal caregivers and training on specific devices needs to be done by the manufacturer. This leads to generic training being given to caregivers by individuals who do not know the patient and who may have very limited knowledge on ALS and how this impacts their use of the device.

The trust I’ve worked in wouldn’t accept the liability for training carers externally. {ALS co-ordinator 4}

Participants highlighted the responsibility of healthcare professionals to educate and inform people with ALS about aspects of care relating to cough and secretion management to ensure that they understand the multifactorial nature of management strategies. They felt that this enabled informed decision-making around their care and supported the ability for them to self-manage their symptoms as able.

Participants highlighted uncertainty about who held overall responsibility for equipment, its training, and the provision of ongoing consumables. This confusion often led to delays in care delivery due to inability to access training or devices.

We are never sure of the process of how to go about getting the equipment and then who is responsible for it. {Respiratory physiotherapist 1}

Healthcare professionals stated that often they were unsure who to approach for medication prescriptions, which caused delays to care. Most participants were not qualified to prescribe medication and highlighted the responsibility of recommending medication to be prescribed by someone who was not an ALS specialist.

### 3.2. Access as a barrier

Participants deemed shared communication vital to support patient care and prevent unnecessary steps in care, which can be time-consuming for healthcare professionals and burdensome for patients.

If we have a cough assist, what settings is it on? Even if they don’t have their own machine with them, what am I setting mine up on so it’s not just trial and error. {Respiratory physiotherapist 2}

Access to patient records throughout the care pathway was a recurrent topic and was consistently highlighted as a major barrier to supporting effective care. This is especially important in cough and secretion management as medication changes can be made as frequently as weekly and numerous people are involved in the management of these problems across numerous organizations. Participants highlighted that they were often unable to access the information they needed to provide optimum care and that a shared point of access to all information about patients would be very useful.

We can’t access GP records and they can’t access ours. {ALS nurse 1}

Participants gave examples of shared communication strategies (e.g., patient passports), but the majority of these were not able to be accessed out of the organization, meaning that shared communication across the pathway was not occurring.

The inequity in access to specialist care and services across the UK and the impact this can have on patient care was consistently highlighted. There were many reasons outlined for this including staffing availability and capacity of specialist care centers, distance required to access this specialist care and appropriateness of care locations. Participants overwhelmingly felt that access to specialist care impacted the patient throughout their disease. They felt that patients with access were much less likely to need acute hospital admissions, and if they did, a care plan ensured good care.

They tend to stay in the community more rather than bouncing back into hospitals with lots of infections and things. {Respiratory physiotherapist 5}

The patients who have seen the respiratory team have a really great plan in place and it’s quite easy to follow and families are on board. {Respiratory physiotherapist 6}

Most specialist ALS care and respiratory specialist centers are located in big cities which are not easy to access or park at, and patients were having to travel long distances to access treatments such as cough assist optimization or Botulinum Toxin. As the disease progressed and physical functioning deteriorated, participants noted that people with ALS were much less likely to access this specialist care, despite this being the time they needed it the most. Participants shared examples of how they had attempted to overcome this barrier using virtual outreach (e.g., video calls) and implementing new local outreach services to support this. One participant described how the implementation of a local Lung Volume Recruitment (LVR) service led to improved, timely access to care due to delays at the specialist respiratory center:

If they were waiting to see the respiratory team there was usually a delay of maybe a week, maybe 2 weeks … whereas we would usually get to them within a couple of days. {Neuro physiotherapist 6}

Procurement of equipment was highlighted as a challenge and a barrier to care. Who provides the devices, the referral routes, and procedures to access these can be a barrier. It was also noted that provision may be limited by funding and skillset.

I am the only person that will deliver cough assists in the community and we only have 3 machines … there’s not enough equipment to go around and I only work 3 days a week. {Neuro physiotherapist 2}

This related to expensive, specialist equipment such as cough assist devices but also access to basic equipment such as peak flow meters which can influence assessment. This had a direct impact on the quality of care being delivered.

We don’t do peak cough flow on anyone and I think that’s because of equipment access. {Respiratory physiotherapist 3}

### 3.3. Relationships as an enabler

The importance of relationships developed between healthcare professionals, people with ALS, their caregivers, outside agencies, and other healthcare professionals was overwhelmingly noted to be a facilitator to care.

Relationships between healthcare professionals were key to providing both a multidisciplinary approach and continuity of care, which was perceived to be a desirable outcome. Participants described how they use existing or past relationships to support training, service development, and share information about patient care, which led to better care outcomes.

I actually worked in area [location close to ALS centre] so I’d got relationships and I went over and spent half a day there. {Neuro physiotherapist 6}

A good relationship between specialist respiratory centers, ALS care centers, and local services was seen as very important. This led to respiratory upskilling in areas and opportunities for supervision. Without this network, participants were not able to offer the quality of care they wished to.

It works well when you get a relationship between your community therapist and tertiary centre … but until you have worked up that network of people it’s really hard. {Neuro physiotherapist 3}

Participants highlighted that relationships between services supported communication when co-location was impossible, and participants described “relief” when they found patients were under centers they had working relationships with as this supported communication. One participant described how lucky she felt to have such a good working relationship with the specialist ALS care and respiratory center and described impacts if this was not the case on both her and patient care. Participants relied on relationships to ensure effective communication between teams. Email was seen as an effective way to communicate about patients, however barriers included relationship development and accessibility of contact details.

I think it would be an absolute disaster to be honest. I’d probably be ringing around like a headless chicken not knowing where to go. {Respiratory physiotherapist 6}

The importance of multidisciplinary team (MDT) working to make good, informed decisions about cough and secretion care and to ensure optimum care was consistently highlighted. It was noted that healthcare professionals are often doing things in their own silos and not stepping back to look at the bigger picture and improved MDT relationships could support this. Participants shared concerns about practice if the right healthcare professionals were not involved.

When we come together, I think we do get better outcomes for the patient. I didn’t work in a MDT service. I worked as a speech therapist on my own and I always thought a cough assist machine would be a great idea if someone had something stuck in their throat to clear it however, now I know that’s incorrect and you could be blowing that material further down into the airway and I’m sure I wasn’t the only SLT to think that. {Speech therapist 1}

Relationships are often built up over time and a high turnover of staff can impact the opportunity to build relationships, which is fatiguing and frustrating for the staff remaining.

We train them and get to know them and then the staff will change and so we seem to be on that constant treadmill of having to refresh. {Neuro physiotherapist 1}

Participants noted that building relationships with patients and carers supported difficult conversations, enabling personal views on their aims, goals, and wishes to be at the center of their care. The anxiety of both patients and carers could potentially be a barrier to care, especially when considering the use of cough devices, but participants felt this could be overcome by developing relationships which led to healthcare professionals being able to provide information at the right time and choose when, how and who to train on certain techniques.

### 3.4. Expectations as a barrier

Discussions around expectations were multi-faceted. Participants felt that the expectations of patients, carers, service managers, and the wider NHS were often not aligned, which became a barrier to care.

A discrepancy was noted between the knowledge of how people with ALS should be managed, and how well participants were able to deliver this service and manage patient expectations.

There’s been a rapid development and rapid change over the last decade or so in how we manage ALS and neuromuscular patients and the expectations of the patients and the service haven’t met or caught up at the same rate. {ALS co-ordinator 4}

Managing the expectations of people with ALS and their caregivers was something that healthcare professionals found challenging and focused on getting to know the family and their needs to prevent this from becoming a barrier to care. People with ALS and their caregivers often expected that a treatment or intervention prescribed by a healthcare professional would “fix” their problems, therefore healthcare professionals identified the need to work with the patient and their family to understand the treatments to align expectations.

Patients just want a quick fix but often it’s lots of appointments and managing expectations. A lot of them want an easy fix. That isn’t going to happen. {Advanced practitioner 1}

Participants’ expectations around the knowledge base and skills of healthcare professionals differed. Care competencies that some participants expected to be a “basic skill” were felt by others to need more structured support with ongoing supervision.

Things like doing PCF [Peak cough flow measurements] and that, it’s basic respiratory skills. None of it is really specialist so those things should be completed wherever the patients are. [Respiratory physio 4]

Participants were clear that they did not expect non-specialists such as GPs to have a broad knowledge base in ALS; however, they were reliant on them to prescribe secretion management medications. Many of these are prescribed outside their licensed indication with limited evidence behind them, therefore communication was deemed to be critical to support this.

If it’s not in the formulary, they often won’t do it at all. {ALS co-ordinator 2}

Participants reported different expectations of the potential value of different treatments and interventions. This could be influenced by their previous experience or personal preference or by the individual patient themselves. This impacted on the options given to patients, and points to discrepancies in care.

I don’t have massive success with cough assists and I find medications a better way to manage it. {Neuro physiotherapist 2}

It was raised that healthcare professional expectations of treatment success can be impacted by the home environment or availability of caregiver support.

But when you go and see someone who’s perhaps in a nursing home, or just perhaps coming in with a carer and doesn’t really have that familial advocate. I will try to give you what might be done at the bare minimum but your expectations are a lot lower and it’s sad that your patient can’t get what they should have. {Respiratory physiotherapist 3}

Participants working within specialist services noted that sometimes there are high expectations of what a specialist service can provide, and this may not always be possible.

There’s such a complex and variable cohort that even when we assess patients in specialist services, we don’t always get it right. I find there is rarely a perfect fix for these patients and often it’s about trying to balance expectations, quality of life and intervention all together. {Speech therapist 1}

Service expectations and expectations of job roles from service managers could limit service development when this was misaligned with the views of staff working on the ground.

I had to fight really hard to be allowed to do a procedure in the community with them saying none of the other physios are skilled. {Neuro physiotherapist 6}

### 3.5. Comprehensive clinical assessment as an enabler

A comprehensive clinical assessment was seen as key to successful cough and secretion management. However, variable practice was noted across regions.

I think that the more nuanced our assessments are and the more MDT information we can put into them, the better outcomes there are for our patients because we are working together. {Speech therapist 1}

The structure of the assessment, and the weighting of patient report vs objective measurements was debated. Overall, a combined approach was perceived to be most valuable to direct treatment inventions most effectively.

I piece it all together, the feeling on their symptoms as well as what the peak cough flow shows. {Neuro physiotherapist 3}

Participants highlighted the need to get a holistic overview considering numerous other factors during the assessment, considering the interplay of symptoms and that these may impact cough and secretion issues. This is done best by utilizing information from numerous MDT members.

Specifically for cough to understand their feeding and swallowing as well as their breathing. I think that [posture and postural management] really affects swallow and this can affect sialorrhea as well. {ALS co-ordinator 1}

The importance of separating the problem into contributing components to understand the underlying physiology and etiology was deemed to be especially important to support prescription of the appropriate treatment.

We see a lot of referrals for cough issues without breaking down whether it’s pulmonary or salivary secretions. It’s about working out what is oral secretions and what is chest secretions and then obviously managing it differently. {Respiratory physiotherapist 3}

This differentiation of oral vs chest secretions and thick vs thin secretions was highlighted as vitally important to support clinical reasoning. Participants highlighted that when a person has both thick and thin secretions, they often don’t fit any of the existing clinical pathways or assessment proformas. Participants agreed that in order to understand the problem, they needed to undertake complex clinical reasoning, drawing together healthcare professionals’ confidence; experience and expertise; understanding pharmacological management strategies; understanding where the secretions originated from and the priority and preferences of the patient.

He is way more worried about his drooling and saliva than he is about his chest. {ALS co-ordinator 2}

Participants overwhelmingly felt that objective markers and measures of cough and secretions were clinically useful but noted that how and when the results are used are important. However, it was noted that this relies on the confidence of healthcare professionals to complete and analyze these assessments and it was noted that picking the right outcome measures and interpreting them to inform treatment strategies requires knowledge and experience.

Confidence in completing outcome measures to actually gain a knowledge of what is the problem because there’s part of it, you identify a problem but you don’t know what to do with it. {Neuro physiotherapist 5}

Participants noted the importance of considering the stage of the disease when choosing appropriate assessment techniques and treatment interventions, highlighting the importance of holistic and individualized management. Participants stressed the need to put patient preferences at the heart of the assessment to support decision-making around treatment interventions.

It is really important that the subject of what the patient is struggling with needs to be key rather than focusing on numbers. {Respiratory physiotherapist 3}

Type of disease and evidence of bulbar dysfunction were also highlighted as key considerations when completing assessment and choosing the best way to assess. Participants felt experience with this patient group was a supportive factor when assessing bulbar impairment.

So many of the patients we see are bulbar and perhaps wouldn’t generate a good peak cough flow.You can tell in their voice if they are not going to generate a good peak cough flow {Respiratory physiotherapist 3}

### 3.6. Suggestions of ways to improve care

Participants felt that the use of a structured assessment tool would support management to avoid things being missed, support clinical reasoning and handover of care between teams.

It would probably serve to standardise or guide interventions at least. {Neuro physiotherapist 4}

I think it’s always good to try and standardise things and to try to give people a prompt and look at where thresholds are. {ALS co-ordinator 4}

Participants felt that a structured assessment tool would be useful to support throughout the pathway. It would offer the opportunity to address inequalities in care provision in situations where specialists were not available, however it was noted that it would not fully solve all problems. It was suggested that any tool developed would have to have the ability to be adaptable to fit within areas with different service provisions.

It’s a really good idea to have a generic tool but it probably just needs to fit the service that it’s in and then it’s still going to lead to an inequality but I don’t think you are ever going to solve that problem. {ALS co-ordinator 4}

It was highlighted that any intervention developed would need to be user-friendly, be adaptable for different stages of the disease and be easy to follow. Participants were asked to state what they would want from this proposed assessment tool, which is summarized in Table 4.

**Table 4.** List of participant suggestions for the tool linked to intervention function.

Tool suggestions	Intervention function	Barriers/enablers this would address
Incorporate all MDT members	Modellingand education	Relationships,responsibility, expectations
Guide assessment and management	Modelling and education	Clinicalassessment, responsibility
A flow sheet with recommendations coming off it *“a decision making tree”*	Education and training	Clinicalassessment, responsibility,expectations, access
A guide to thinking and management	Education and training	Clinical assessment, responsibility, expectations
Support clinical reasoning around bulbar dysfunction	Education and training	Responsibility, access, clinical assessment
Be standardized across the UK	Enablement	Access, expectations
Facilitate conversations and communication about patients	Environmental restructuring	Relationships, access, responsibility
Use of technology to support	Environmental restructuring	Access, clinical assessment, relationships
Support healthcare professionals to access specialist services	Environmental restructuring	Access, expectations

MDT = multidisciplinary team.

Participants noted the importance of consistency and consistency of language which would support to facilitate communication and conversations and felt it would benefit from being linked with an organization such as the MNDA.

Everyone singing off the same hymn sheet. {Neuro physiotherapist 7}

...facilitate those wide conversations across professions. {ALS nurse 3}

Table 4 lists suggestions participants had for the tool linked to the behavior change wheel intervention functions [31].

## 4. Discussion

Cough and secretion management is complex and multifaceted, however it is vitally important to promote quality of life and potentially improve survival for people living with ALS. Following on from a national survey [35], which found that numerous professionals provide care for these problems with low confidence levels, these focus groups allowed deeper discussions around the barriers and enablers to care from the perspectives of healthcare professionals, mapped to behavior change frameworks. Mapping to these frameworks allows identification of potential behavior targets when future interventions are developed.

Cough and secretion management benefits from a holistic and structured approach requiring multiple different specialties to work together to achieve the best outcomes. Therefore, defining and allocating roles and responsibilities for all these different components can be challenging but is also key to success. It appears that any intervention development in the future must consider this and consider how to define the key components which might lead to change and who contributes to these processes. Five themes were generated from inductive thematic analysis. Although these were classed as either a barrier or an enabler, it must be noted that all can be both, and there may be some fluidity between this depending on the context. The data collected from these focus groups mapped onto all domains of the COM-B and all TDF domains except reinforcement, highlighting the number of behaviors involved.

The domains with the most relevance were environmental context/resources (TDF) and physical capability (COM-B). These relate to access to services, funding, different commissioning structures, and resources. Participants reported, as found in other recent studies, that often practical aspects to care, such as access to patient records, were the biggest barriers [39,43]. The importance of informal caregivers to support communication, care, and implement management strategies was emphasized in this study and by other recent ALS studies [43,49] and other studies on cough augmentation in neuromuscular disease [50]. This is similar in NIV care, where studies have shown that people who are married with informal caregiver support were much more likely to be adherent to their NIV program [51,52].

There are numerous professionals involved in cough and secretion care in ALS therefore who takes responsibility for all aspects of care and education appear to be key in ensuring that care is delivered optimally. This links with the domains of social professional role and identity (TDF) and reflexive motivation (COM-B). It was highlighted that staff often *“don’t know what they don’t know”* and that there is a role for ALS specialists to guide this upskilling using mentorship and education, which would lead to improved care. This was noted by Ackrivo [53] who highlighted the lack of specific training and education programs for healthcare professionals in ALS care, with limited mentors with the clinical experience to support this. This meant that there was a significant burden on these staff with the clinical experience and skills. Walls [54] highlighted the psychological impact this can have on healthcare professionals including burnout, frustration, and anxiety, which can lead to increased turnover of staff. Access to healthcare professional training and education is imperative to be able to support healthcare professionals in their day-to-day work to share practice and disseminate knowledge. The development of these “local champions” could be crucial to support the implementation of new interventions [55,56]. Defining who is responsible for each implementing each component of a novel intervention is key to ensuring its effective implementation and success. Accessing specialist services who have specialist care skills such as Botulinum toxin were highlighted as a key barrier in previous UK surveys [34,57]. Also, how healthcare professionals can gain these specialist care skills such as prescribing or injecting in their specific areas was also discussed as an ongoing barrier. This may differ in different parts of the country due to service set up including co-location and geographical spread leading us to conclude that a one size fits all approach would not work. Local adaptations and prioritization should be used to support implementation of any future interventions.

The importance of specific knowledge of ALS and ability to be able to develop a scientific rationale when completing an assessment was deemed to be vitally important to providing good care. This links with the domains of knowledge (TDF), skills (TDF), and physical capability (COM-B) highlighting the importance of knowledge and skills in delivering good-quality cough and secretion care. Participants felt that a thorough assessment with sound clinical reasoning was the primary factor to facilitate provision of appropriate ongoing interventions. They felt that a standardized assessment to guide interventions would support equity of access to care. Guidelines [20,29,30] often discuss assessment strategies in silos and do not discuss how cough assessments are linked to saliva and secretion assessments. Clinical research in cough and secretion management is often heterogeneous to allow for a meta-analysis [37] meaning that many recommended assessments were not based on an ALS population such as MI-E titration protocols [24,25]. The use of objective assessment tools was also impacted by numerous factors such as training, skills, availability of equipment, service set up [57]. The ever-changing structure of the NHS and navigating commissioning differences between the different newly appointed, regional Integrated Care Boards means that access to care remains inequitable and changeable [58].

Responsibility for medication prescribing was repeatedly highlighted. It must be noted that there was only one healthcare professional in all four focus groups who was licensed to prescribe medication. Currently in the UK, certain professionals can be trained to become registered medication prescribers including nurses, physiotherapists, paramedics, and dieticians, however there are still groups such as speech and language therapists who are not currently allowed to become prescribers. Graham-Clarke [59] discussed how non-medical prescribing can be more easily adopted into practice where it can form part of the overall care of the patient, however there is limited data on the impacts on professional groups that are unable to become non-medical prescribers. The value of prescribers embedded within these teams was not sufficiently explored in the focus groups.

Relationships with healthcare professionals, outside agencies, and patients and their caregivers were noted to be important enablers of care. It has been highlighted that relationships can also support the implementation of new interventions or pathways [60]. This is linked with the alignment of expectations. Overall, it was felt that the misalignment of these presented a barrier to care. Healthcare professionals described fear that they may not be able to meet the patients’ expectations impacting the relationship between themselves and the patient/caregiver, this was echoed in a similar study around NIV in ALS [61]. In this study, we did not explore how individual healthcare professional characteristics such as physical health, mental health and mood may have impacted care, but this is something to consider for future work.

### 4.1. Strengths and limitations

This is the first study to explore the barriers and facilitators to cough and secretion management in ALS in the UK. It combined both inductive and deductive analysis to identify themes emerging and also behaviors involved which will allow for future interventions to be targeted. Participants were recruited from a variety of locations and settings. However, a convenience sample was recruited. Participants represented a range of professions, however there was a larger number of physiotherapists in the group. There was a lack of representation from GPs, neurologists or respiratory physicians. It is possible that the participants in this study were those most engaged and interested in cough and secretion management care. Additionally, the views of healthcare professionals not captured here could strengthen understanding of how cough and secretion management occur in practice.

## 5. Conclusion

The management of cough and secretion issues in ALS is variable. This study identified several key factors that impact delivery of cough and secretion management care. These include access to both equipment and specialist care, the responsibilities and roles of each member of the MDT, and the relationships and expectations between ALS services, professional groups, and people with ALS and their caregivers. A thorough, holistic clinical assessment with clear clinical reasoning, access to education to improve knowledge and skills and access to highly specialist support as and when required were identified as key enablers to care. The findings of this study can inform improvements in care both within the UK and internationally and future research should seek to obtain the views of people living with ALS and their caregivers, to identify whether these align with the views of healthcare professionals gathered in this study. In turn, this will support the development of complex interventions in cough and secretion management in ALS which could be implemented universally.

## Supplementary Material

Supplemental Material

## Article highlights

- ALS is a rapidly progressive neurological condition with no cure
- Cough and secretion issues are common, multifactorial, and complex to manage but there is limited research exploring healthcare professional’s experiences managing these
- Focus groups held with UK healthcare professionals identified five themes as barriers or enablers to providing care:
  - Enablers were responsibility, relationships, and clinical assessment
  - Barriers were access and expectations
- Numerous behaviors were identified by mapping to behavior change frameworks including environmental context, education, and skills
- Care within the UK remains variable and is impacted by access to equipment and specialist care and the responsibilities and relationships of multidisciplinary team members.
