# Supplementary material for: Exploring mechanisms of behavior change for healthcare professionals in cough and secretion management in ALS
Source: Neurodegener Dis Manag. 2025 May 20;15(4):149–60. doi: 10.1080/17582024.2025.2506954 (PMC12296054; doi:10.1080/17582024.2025.2506954)
Supplement: Supplemental Material [file INMT_A_2506954_SM0721.zip › suppl_data/Supplementary material 1 - Survey summary data given to participants during the focus groups.pdf]

INITIAL THEMES FROM SURVEY

Better outcomes in MND

BARRIERS TO CARE

- Electronic records communication
- Quality of documentation
- Identifying team members involded and being able to contact them
- Team relationships
- Duplication
- Knowledge base/skill set
- Access to equipment
- Patient expectations
- Carer support
- Time and funding
- Lack of evidence base

FACILIATORS OF CARE

- Shared electronic records
- Patient held record
- MND passport/hub/portal
- MDT communication/integration
- Joint MDT clinics
- Team relationships
- Timely/early access to intervention
- Knowledge base/skill set
- Access to equipment
- Access to specialists/experts
- Carer support
- Research/evidence

BIGGEST CHALLENGES

- Bulbar involvement
- Throat vs chest secretions
- Speed of progressing
- Limited response to pharmacological management
- Mixed thick vs thin secretions
- Access to suction machines
- Local community follow up
- Accessing specialist services
- Carer dependence
- Number of HCPs involved

LEARNING NEEDS

- Bulbar impact on respiratory function
- Research/evidence base
- Saliva/secretion management
- Cough augmentation
- Respiratory muscle training
- Swallow and impacts
- Holistic care
- Reflux
- Use of nasendoscopy in decision making
