# Supplementary material for: Exploring mechanisms of behavior change for healthcare professionals in cough and secretion management in ALS
Source: Neurodegener Dis Manag. 2025 May 20;15(4):149–60. doi: 10.1080/17582024.2025.2506954 (PMC12296054; doi:10.1080/17582024.2025.2506954)
Supplement: Supplemental Material [file INMT_A_2506954_SM0721.zip › suppl_data/Supplementary material 3 - COM-B and TDF framework mapping.docx]

**Supplementary material 3:** COM-B and TDF framework mapping

| **COM-B component** | **Theoretical domains framework** | **Influencing factors** | **Quotes** | **Intervention function** | **Individual Behaviour change technique (BCT)** |
| --- | --- | --- | --- | --- | --- |
| Psychological capability | Knowledge | - Knowledge of measures to inform assessment  - MDT knowledge of ALS  - Imparting and sharing knowledge  - people with ALS knowledge  - Informal caregiver knowledge  - Expected levels of knowledge | *“They don’t know what they don’t know” {MND nurse 2}*  *“The big lack of understanding about the MND itself” {Respiratory physiotherapist 2}* | - Education  - Training  - Enablement  - Modelling | - Information about health consequences  - Instruction on how to perform the behaviour  - Mental rehearsal of successful performance  - Focus on past success  - Exposure |
|  | Memory, attention and decision processes | - How decisions are made by different team members  - Feelings of responsibility | *“If one of us wasn’t in that’s when someone would really struggle in the management of these patients…they could provide safe care but it certainly wouldn’t be specialist care” {Respiratory physiotherapist 1}* | - Modelling  - Enablement  - Training | - Graded tasks  - Re-attribution  - Comparative imagining of future outcomes  - Problem solving  - Framing or reframing |
|  | Behavioural recognition | - NHS management is reluctant to change interventions  - Being proactive – how to support this in current systems  - How to implement new treatment options | *“You need the staff to be inclined to do that” {MND co-ordinator 1}*  *“I had heard about LVR bag and went to *** to upskill and started using it more with my patients because I was seeing a huge gap at that point” {Neuro physiotherapist 6}* | - Restriction  - Modelling  - Enablement  - Persuasion  - Training | - Demonstration of the behaviour  - Information about health consequences  - Incentive  - Social support (practical) |
| Physical capability | Skills | - Clinician experience and expertise to analyse assessment and results  - Knowing when to change management  - Prescribing  - Both skills to give treatment and know when to refer on  - Specialist skills such as nasendoscopy and MI-E | *“I feel like I have a good understanding of the medication but I’m not a prescriber” {MND co-ordinator 1}*  *“Confidence completing outcome measures to actually gain a knowledge of what is the problem because there’s part of it, you identify a problem and you don’t know what to do with it” {Neuro physiotherapist 5}* | - Modelling  - Enablement  - Environmental restructuring  - Training | - Instruction on how to perform the behaviour  - Social support (practical)  - Exposure  - Associative learning  - Adding objects to the environment |
| Social opportunity | Social influences | - Face to face vs virtual assessments  - Gaining trust of patient  - Building rapport with patient  - Trust within the MDT | *“I can outreach virtually but it’s not quite the same” {Neuro physiotherapist 3}*  *“When you go and see someone who’s perhaps coming in with a carer who doesn’t have that family advocate. I will try and give you what might be done, the bare minimum but your expectations are a lot lower and it's sad that your patient can’t get what they should have” {Respiratory physiotherapist 3}* | - Restriction  - Persuasion  - Enablement | - Action planning  - Problem solving  - Restructuring the social environment  - Social comparison  - Social support (emotional) |
| Physical opportunity | Environmental context/resources | - Other risk factors such as swallowing, feeding, diet, upper limb and ventilation  - Bulbar impairment  - Postural management and mobility  - Care pathways  - Accessibility to specialists  - MDT working  - Local management pathways  - Access and funding  - Equality of commissioning across the UK  - Resource to train on devices (e.g. carers)  - Access to patient records  - Reliance on others to share information  - Access to equipment  - Patient passports  - Availability of staff  - Service structure  - Lack of evidence-based guidelines  - Access to training | *“We don’t do peak cough flow at *** and I think that is because of equipment access” {Respiratory physiotherapist 3}*  *“It’s the capacity, it’s a staffing issue” {MND nurse 1}*  *“We are never sure of the process of how to go about getting equipment and then who is responsible for it” {Respiratory physiotherapist 1}* | - Environmental restructuring  - Education  - Training  - Enablement  - Persuasion  - Restriction | - Instruction on how to perform the behaviour  - Behavioural practice/rehearsal  - Adding objects to the environment  - Information about health consequence  - Prompts/cues  - Social comparison |
| Reflective motivation | Social professional role and identity | - Importance of language used  - Whose role it is  - Who is taking the risk  - Role and responsibilities not clear  - No clear lead | *“I feel quite uncomfortable being the sole practitioner holding that risk” {MND co-ordinator 1}*  *“Who is the lead in taking that patient’s management forwards” {MND nurse 3}* | - Environmental restructuring  - Education  - Enablement  - Modelling | - Framing or reframing  - Identity associated with changed behaviour  - Comparative imagining of future outcomes  - Information about health consequences |
|  | Beliefs about capability | - When to refer to specialist services  - Roles – who should do what  - Staff “don’t know that they don’t know” | *“They are not sure who to escalate to” {Speech therapist 2}*  *“When we come together, I think we do get better outcomes for the patient” {Speech therapist 1}* | - Education  - Training  - Enablement | - Instruction on how to perform the behaviour  - Social support (unspecified)  - Feedback on outcome(s) of behaviour |
|  | Optimism | - Role modelling  - Post COVID-19 NHS landscape | *“I have put together a respiratory screening tool that is simple and goes through all the signs and symptoms” {MND co-ordinator 1}* | - Modelling  - Enablement | - Rewarding completion  - Demonstration of the behaviour  - Restructuring the social environment |
|  | Beliefs about consequences | - Consequences of working outside of professional boundaries but also consequences of not doing this on direct patient care | *“So, if we are not in, I don't mind the phoning because I would rather have that conversation” {Neuro physiotherapist 1}* | - Education  - Training  - Enablement  - Modelling  - Restrictions | - Habit reversal  - Over correction  - Behavioural practice/rehearsal  - Social support (unspecified)  - Pros and cons |
|  | Intentions | - Examples of services initiated due to one team member | *“I had to fight really hard to be allowed to do a procedure in the community” {Neuro physiotherapist 6}* | - Modelling  - Training  - Enablement  - Restrictions | - Identification of self as role model  - Social support (unspecified)  - Information about health consequences |
|  | Goals | - Aims, goals and wishes of the patient  - Patient centred approach | *“Their aims, goals and wishes” {MND coordinator 1}*  *“It is really important that the subject of what the patient is struggling with need to be key rather than focusing on numbers” {Respiratory physiotherapist 3}* | - Enablement  - Education | - Restructuring the social environment  - Prompts/cues  - Exposure  - Associated learning |
| Automatic motivation | Emotion | - Difficult conversations  - Guilt when unavailable  - Anxiety of carers  - Ceilings of care  - Healthcare professional anxiety of unknown devices or treatment options  - No formal psychological support for staff  - Staff feeling isolated and scared | *“Sometimes its quite a lonely place when you are in clinic on your own trying to give that confidence to a patient that you are going to give them something that will make them better” {Advanced practitioner 1}*  *“It's psychologically very difficult. It's very difficult” {Respiratory physiotherapist 1}* | - Enablement  - Environmental restructuring | - Valued self-identify  - Focusing on past successes  - Social support (emotional)  - Information about emotional consequences |

Table legend: COM-B = Capability, opportunity and motivation behaviour model, TDF = Theoretical Domains Framework, MDT = multidisciplinary team, NHS = National Health Service, MI-E = Mechanical insufflation-exsufflation, COVID-19 = SARS-CoV-2 virus pandemic

Table adapted from Cane et al (2012) and Keyworth et al (2019)
